# Intra-tumoral delivery of functional ID4 protein via PCL/maltodextrin nano-particle inhibits prostate cancer growth

**DOI:** 10.18632/oncotarget.10953

**Published:** 2016-07-30

**Authors:** Maxwell Korang-Yeboah, Divya Patel, Derrick Morton, Pankaj Sharma, Yamini Gorantla, Jugal Joshi, Perri Nagappan, Ravi Pallaniappan, Jaideep Chaudhary

**Affiliations:** ^1^ College of Pharmacy, Mercer University, Atlanta, GA, USA; ^2^ Center for Cancer Research and Therapeutic Development, Clark Atlanta University, Atlanta, GA, USA

**Keywords:** ID4, prostate cancer, tumor suppressor, nano-carrier, protein delivery

## Abstract

ID4, a helix loop helix transcriptional regulator has emerged as a tumor suppressor in prostate cancer. Epigenetic silencing of ID4 promotes prostate cancer whereas ectopic expression in prostate cancer cell lines blocks cancer phenotype. To directly investigate the anti-tumor property, full length human recombinant ID4 encapsulated in biodegradable Polycaprolactone/Maltodextrin (PCL-MD) nano-carrier was delivered to LNCaP cells in which the native ID4 was stably silenced (LNCaP(-)ID4). The cellular uptake of ID4 resulted in increased apoptosis, decreased proliferation and colony formation. Intratumoral delivery of PCL-MD ID4 into growing LNCaP(-)ID4 tumors in SCID mice significantly reduced the tumor volume compared to the tumors treated with chemotherapeutic Docetaxel. The study supports the feasibility of using nano-carrier encapsulated ID4 protein as a therapeutic. Mechanistically, ID4 may assimilate multiple regulatory pathways for example epigenetic re-programming, integration of multiple AR co-regulators or signaling pathways resulting in tumor suppressor activity of ID4.

## INTRODUCTION

Inhibitor of differentiation 4(ID4), a helix loop helix transcriptional regulator [1] has emerged as a major tumor suppressor in prostate cancer (PCa) [2]. EZH2 dependent epigenetic silencing of ID4 is observed in majority of PCa with increasing grade [3]. Genetic ablation of ID4 in non-tumorigenic PCa cell lines such as androgen sensitive LNCaP cells promotes tumorigenicity and castration resistance phenotype both *in vitro* and *in vivo* [4]. Alternatively over-expression of ID4 in tumorigenic DU145 cells that lack native ID4 attenuates their growth and promotes androgen receptor expression [5]. Studies have demonstrated that genetic ablation of Id4 in mice (Id4−/−) leads to attenuated prostate development and PIN lesions as early as 6 weeks post-partum which is associated with a pro-neoplastic phenotype (Pten-, Id1+, pAkt+, Ki67+, Nkx3.1-) [6]. These studies suggest that ID4 deficiency is a major event in PCa. Thus re-introducing ID4 in PCa cells could be a potential PCa therapeutic approach.

ID4 promotes p53 [7] and androgen receptor (AR) transcriptional activity [4], promotes senescence independently of p16 and Rb and induces the expression of multiple cyclin dependent kinase inhibitors (CDKNI) [5, 8]. Lack of the DNA binding domain in ID4 [1] suggests that most of its biological activity could be due to protein-protein interactions. In fact, recent studies have shown that ID4 can interact and functionally inhibit the activity of bHLH proteins TWIST1 [9] and the ID paralogs ID1, ID2 and ID3 [10] which are up-regulated in PCa [11, 12].

The intracellular proteins, specifically tumor suppressor proteins whose expression is either lost/and or mutated (e.g. PTEN, Rb, and p53) have crucial roles in either initiation and/or progression of cancer [13]. Thus restoring the tumor suppressor activity either through genetic, protein delivery (whole proteins, small peptide mimetics) and/or epigenetic modifications is considered as the hall marks of cancer therapeutics [14, 15].

As “information rich” macromolecules, protein/peptide drugs offer incredible selectivity, high target specificity, low toxicity and associated unwanted side effects. It is therefore not surprising that proteins/peptides are the ultimate drugs of choice for treatment of various diseases including cancer. In spite of these advantages, the use of proteins or peptides to treat cancers is a challenging approach due to poor stability and low cellular permeability. Consequently, multiple approaches are currently being explored to protect the biological activity of proteins, aid in intracellular transport and enhance target specificity through engineered protein modifications and delivery methods (Reviewed in [15, 16]). Success has been achieved with proteins/peptide drugs and antibodies with some of them in clinical trials [17, 18]. Advances in nanotechnology has made it possible to encapsulate proteins/peptides to minimize degradation and tissue/site targeting through appropriate modifications resulting in increased uptake and intracellular activity (reviewed in [19, 20]). Recent studies have in fact demonstrated successful targeted delivery (receptor mediated endocytosis) of nano-encapsulated p53 into cancer cells resulting in apoptosis [21].

More recently, the use of biodegradable nanocarriers (NC) such as PLGA [20] and polycaprolactone (PCL)- maltodextrin (MD)[22] has gained significant attention to deliver protein intracellularly. The PCL-MD based NC are particularly interesting since both are FDA approved with minimum cytotoxicity [22, 23]. PCL-MD based NC can efficiently deliver large macromolecules such as BSA into the PCa cells while maintaining the secondary structure [22]. Building upon these preliminary observations, we developed the bio-degradable PCL-MD NC encapsulating recombinant ID4 (ID4NC). The tumor suppressor activity of ID4NC was investigated *in vitro* and *in vivo* in castration resistant PCa cell line LNCaP in which ID4 was knocked down via gene specific shRNA. Our results suggest that ID4NC delivered functional ID4 into the cells in which ID4 was knocked down at the gene level, resulting in growth inhibition both *in vitro* and *in vivo*.

## RESULTS

The detailed characterization of PCL-MD NC, including internalization mechanism, kinetics, biocompatibility with various PCa cells lines and sub-cellular localization has been reported earlier [22]. In this study we focused on investigating the therapeutic potential of PCL-MD NC with encapsulated ID4.

### ID4 is encapsulated in PCL-MD NCs

**Figure 1:**
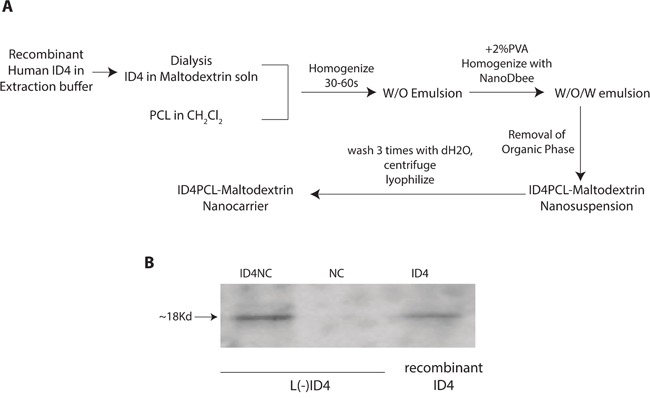
**A.** Schematic flow chart for the synthesis of PCL-MD NC and subsequent encapsulation of recombinant ID4. The details of the process are outlined in materials and methods. **B.** Western blot analysis of ID4. 500 ng of recombinant ID4 (ID4, positive control) was used in an immunoblot analysis to demonstrate that ID4 is released from the nano carriers (ID4NC). No ID4 was detected in the unloaded/blank nano carriers (NC). The blot is represented of more than 4 experiments.

Immunoblotting of L(-)ID4 cells demonstrated that ID4 was released from the NCs encapsulated with ID4 (ID4NC) as opposed to blank NCs (Figure 1B). These results indicated successful ID4 encapsulation process outlined in Figure 1A.

### Uptake of nano-carrier encapsulated ID4

**Figure 2:**
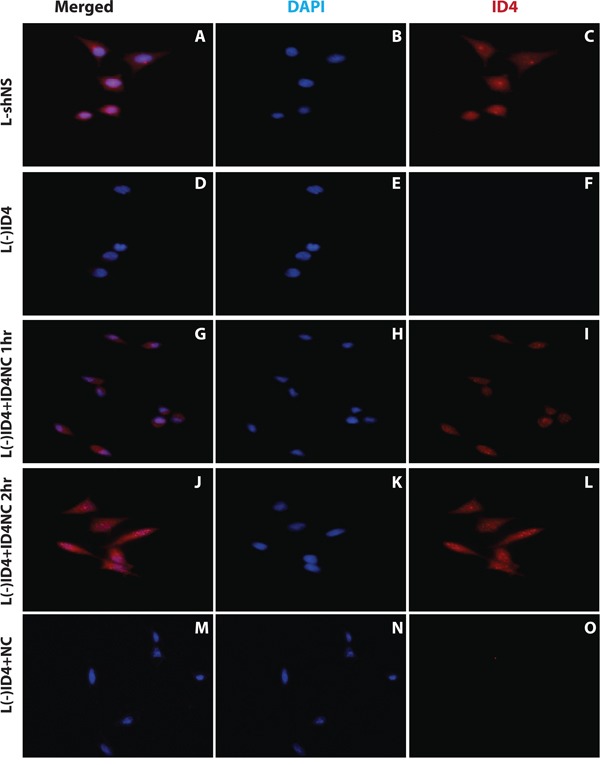
Immuno-cytochemical localization of ID4 in LNCaP cells The two channels showing blue DAPI (nuclear, B, E, H, K, N), red (ID4 protein specific, C, F, I, L, O) staining are shown separately. Both these channels are merged (A, D, G, J and M) to show the cellular localization of ID4 in LNCaP cells: transfected with non-specific shRNA (L(-)shNS: A, B and C), stably transfected with ID4 specific shRNA (L(-)ID4: D, E and F), L(-)ID4 cells treated with ID4 nano carrier for 1hr (L(-)ID4+ID4NC 1hr: G, H and I), L(-)ID4 cells treated with ID4 nano carrier for 2hr (L(-)ID4+ID4NC 1hr: J, K and L) and L(-)ID4 cells treated with blank nano carrier for 2hr (L(-)ID4+NC: M, N and O). The figures are representative of at least three experiments.

The L(-)shNS cells expressed ID4 (Figure 2A and 2C) whereas L(-)ID4 lacked detectable ID4 protein (Lack of red staining in L(-)ID4, Figure 2D and 2F), confirming our earlier studies [4].

Incubation of ID4NC with L(-)ID4 cells led to increased intracellular levels of ID4. The expression of ID4 was observed in both the cytoplasm and nucleus that was consistent with the native ID4 expression pattern (compare Figure 2G, 2I, 2J, 2L with 2A and 2B). In contrast, no ID4 expression was observed in the L(-)ID4 cells incubated with NC alone (Figure 2M and 2O). In another set of experiments, we incubated L(-)ID4 cells with ID4 alone. In this setting, no ID4 was observed in the L(-)ID4 cells after 1 or 2 hrs of incubation. The ID4 ICC in these cells was similar to that shown for L(-)ID4 (Figure 2D and 2F) hence the data is not shown. These results confirmed that uptake of ID4 in L(-)ID4 cells is dependent on its encapsulation in NC.

### ID4NC blocks survival of L(-)ID4 cells

**Figure 3:**
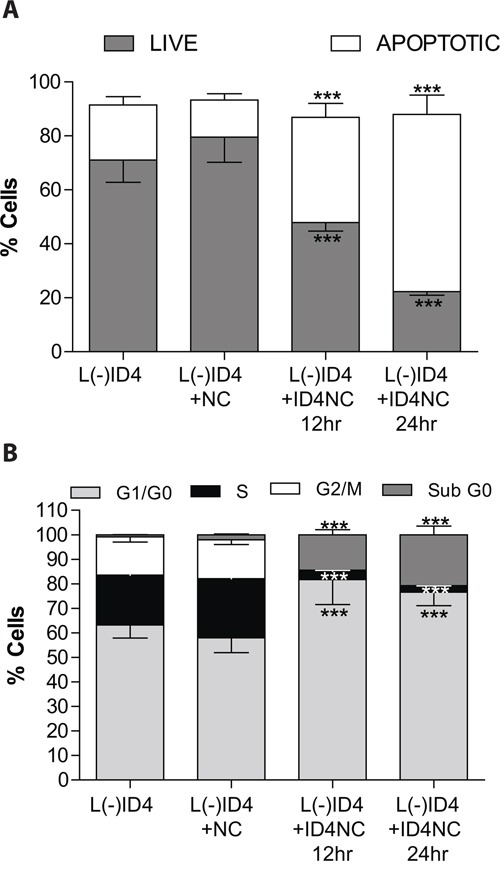
Effect of ID4NC on apoptosis and cell cycle in L(-)ID4 cells The cells were treated with blank nano carrier (L(-)ID4+NC) or with ID4 nano carrier (L(-)ID4+ID4NC) for 12 or 24 hrs. Percent L(-)ID4 cells undergoing apoptosis **A.** Was determined after staining the cells with Annexin V–Alexa 488 and PI and with PI alone for cell cycle analysis **B.** The histogram shows % live and apoptotic cells (A) and the percentage of cells in G0-G1, S, and G2-M phases (B). The graphs represent mean±SEM from three replicates and at least three independent experiments. (***: P<0.001 by student's t-test).

The NCs are biocompatible with normal prostate epithelial cells and PCa cell lines [22]. Therefore a significant change in the number of apoptotic cells in L(-)ID4 with or without NC for at least 24 hrs was not expected (Figure 3A). However, in the presence of ID4NC for 12 and 24hrs, a significant increase in the number of apoptotic cells (12hrs: 39.1±5.2, 24hrs: 65.66±7.2, P<0.001) as compared to untreated (20.48±3.1) or treated with NC alone (13.82±2.3) was observed (Figure 3A). An ID4 dependent increase in apoptosis was also associated with a concomitant decrease in the number of live cells (Figure 3A). These results are consistent with our earlier studies showing that over-expression of ID4 in DU145 cells promote apoptosis [8] whereas silencing of ID4 in LNCaP cells attenuates apoptosis [4].

Loss of ID4 in LNCaP cells promotes cell cycle by shortening the G1/G0 phase [4]. Restoring ID4 expression through ID4NC dependent intracellular delivery significantly increased G1 arrest in L(-)ID4 cells (81.7±10.2 and 76.55±5.4% at 12 and 24hrs respectively, P<0.001) as compared to untreated (63.2±5.3) or treatment with NC alone (57.9±6.1) (Figure 3B). An increase in the population of cells in subG0 phase, a crude estimation of apoptotic cells confirmed increased in apoptosis after ID4NC treatment as compared to controls. A significant decrease in the fraction of cells in S- (3.2±0.5 and 2.1±0.4: 12 and 24 hrs respectively) and G2/M phase (0.6±0.08 and 0.46±0.66: 12 and 24 hrs respectively) further suggested that the cells are primarily arrested in the G1 phase following treatment with ID4NC.

### ID4NC blocks, invasion and anchorage independent cell growth

**Figure 4:**
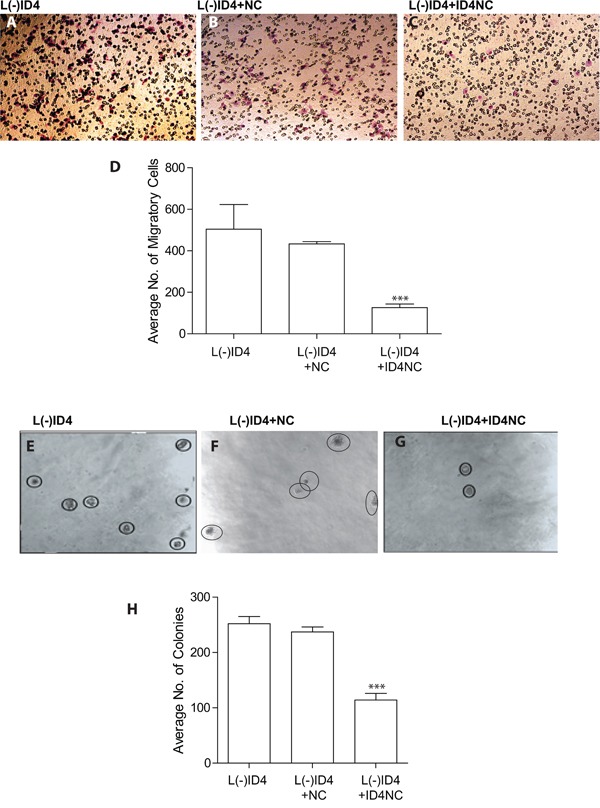
Motility, invasion and anchorage independent growth of L(-)ID4 cells treated with ID4 nano carrier (ID4NC) **A-C.** The invasive capacity of untreated cells (A), or cells treated with blank nano carrier (L(-)ID4+NC) or with ID4 nano carrier (L(-)ID4+ID4NC) across Matrigel. The number of cells migrated across the Matrigel membrane (Pink, representative image shown) were stained (HEMA2 stain set, Fisher) and counted. **D.** The number of cells migrated across the Matrigel membrane (shown in panels “A-C”) were counted and expressed as mean ±SEM (n=3, *P<0.001). **E-G.** Anchorage independent growth of untreated cells (E), or cells treated with blank nano carrier (L(-)ID4+NC, F) or with ID4 nano carrier (L(-)ID4+ID4NC, G) was assessed in a soft agar assay (representative images). The visible colonies are encircled. **H**. The number of independent colonies were counted (panels “E-G”) and expressed as mean+SEM (n=3, ***P<0.001).

We have shown earlier that knockdown of ID4 in LNCaP cells (L(-)ID4) promotes invasion and anchorage independent cell growth [4]. Restoring ID4 expression in L(-)ID4 cells with ID4NC decreased invasion through matrigel by at least 4 fold (p<0.001) as compared to L(-)ID4 cells (Figure 4A-4C and 4D). The anchorage independent growth of L(-)ID4+NC cells in soft agar demonstrated approximately 2 fold (p<0.001) decrease in number of colonies as compared to L(-)ID4 and L(-)ID4+NC cells used as controls (Figure 4E-4G and 4H). These results suggested that gain of ID4 attenuates invasion and anchorage independent growth, the hallmarks of an aggressive cancer, supporting its role as a tumor suppressor.

### ID4 PCL-MD NCs blocks tumor growth of subcutaneous xenografts *in vivo*

The L(-)ID4 cells are castration resistant and readily forms tumors in intact and castrated nude mice [4]. In this study, the L(-)ID4 cells were subcutaneously injected into the flanks of intact SCID beige male mice to investigate the effect of ID4NC on tumor formation. After six weeks of initial subcutaneous delivery of cells, the ID4NC were injected intratumorally, twice weekly for additional three weeks. The experiment was subsequently terminated, tumors extracted weighed and volumes measured.

**Figure 5:**
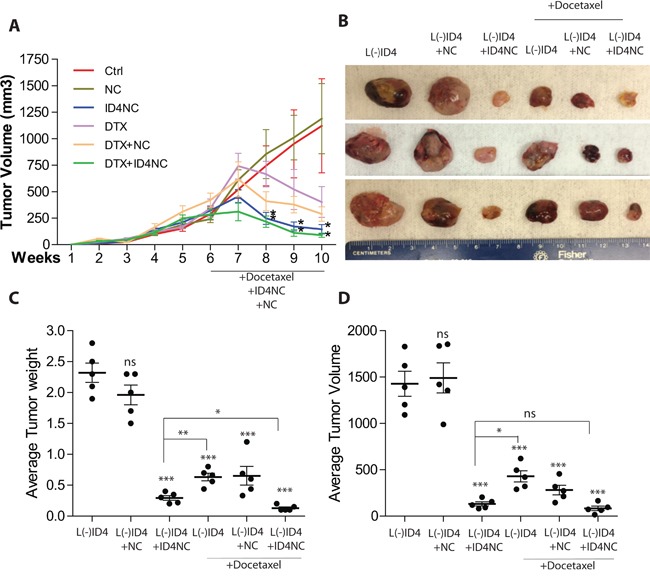
ID4 nano carriers blocks tumor growth *in vivo* The L(-)ID4 cells in Matrigel were injected in the right flank of male SCID Beige mice. After 6 weeks of growth, the tumors were either left untreated (control, Ctrl, L(-)ID4) or treated (intratumoral injections) with blank nano carrier (+NC) or ID4 nano carrier (+ID4NC) with or without DTX twice weekly for 4 weeks. The mice were terminated after 10 weeks of initial subcutaneous implantation of cells. **A.** The volume of the tumors was measured weekly (expressed as mm^3^, mean±SEM, n=6 per group, *: p<0.05) **B.** The representative xenograft images are shown at the end of the study. **C** and **D.** Respective weight and volume (mean±SEM, n=6) of the tumors after excision from the mice. The comparisons are between treatments as compared to L(-)ID4 cells. Respective within treatment comparisons are shown by lines.(ns: non-significant, *: p<0.05, **: p<0.01, ***: p<0.001).

After a latent period of approximately 3 weeks an accelerated tumor growth was observed in SCID Beige mice injected subcutaneously with L(-)ID4 cells (Figure 5A). The mean volume of tumors at the end of the six weeks and before starting treatments was approximately 247mm^3^. Tumor regression was apparent within two weeks of the treatment, particularly in xenografts receiving ID4NC and ID4NC+ DTX (P<0.01) (Figure 5A). The tumor volumes were not different from respective controls in mice receiving NC alone (Figure 5A). Following excision of the tumors, no significant tumor regression was observed in tumors injected with NC alone (weight: 1.96±0.16gm, volume: 1616.572±367mm^3^, Figure 5B) as compared to tumors in mice receiving no treatment (weight: 2.3±0.156gm, volume: 1427±134.7mm^3^, Figure 5B, 5C and 5D). These results suggested that NC alone had no effect on tumor growth over the treatment period. Surprisingly, a highly significant tumor regression (weight: 0.294±0.038gm, volume: 130.9±22.4mm^3^, P<0.001) was observed following intratumoral delivery of ID4NC in all mice (Figure 5B, 5C and 5D). The total body weight between all treatment groups was not significantly different (data not shown) and all mice survived the entire duration of the experiment suggesting no adverse toxicity overall.

Based on the observations that ID4 promotes multiple anti-cancer pathways such as apoptosis and androgen receptor activity, we therefore tested the hypothesis that a combination of DTX, the first line chemotherapy for advanced prostate cancer [24, 25] and ID4-NC could have a synergistic effect on the regression of tumors than when treated with either DTX or ID4NC alone.

Intratumoral delivery of DTX alone also significantly reduced the tumor size (weight: 0.458±0.07gm, volume: 351.7±47.2) as compared to L(-)ID4 control and blank PCL-MD NC (p>0.001) (Figure 5B, 5C, 5D). However, the size of DTX treated tumors was significantly higher (P<0.01, Figure 5) as compared to the ID4NC treated tumors suggesting that ID4NC was therapeutically more active in reducing tumor burden as compared to DTX alone. The DTX+ ID4NC combination significantly (P<0.05) reduced the tumor weight but the tumor volumes were similar to ID4NC treated tumors (Figure 5B, 5C). Overall, these results suggested that ID4NC alone is sufficient to reduce tumor size as compared to DTX alone or in combination.

### Xenograft morphology

The H&E staining of L(-)ID4 xenografts revealed a typical morphology of a tumor tissue with large nuclei and expansive vascularization [26]. The morphology of L(-)ID4 tissue (Supplemental Figure S1A) was similar to L(-)ID4+NC (Supplemental Figure S1B) suggesting that the blank NC had no observable effect overall. In general no pleomorphic nuclei were observed suggesting that the cells were clonally derived (from L(-)ID4 cells). No major differences were observed between the morphology of DTX alone or DTX +NC xenograft tissues.

### Tumor apoptosis

**Figure 6:**
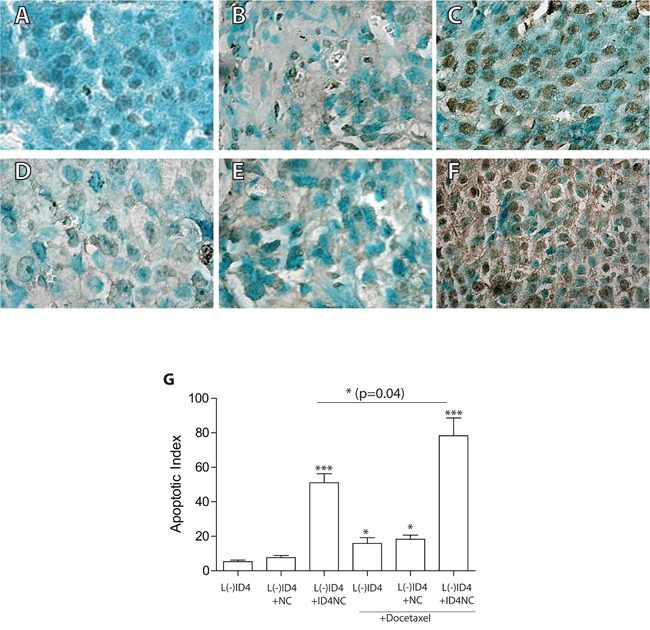
Apoptosis in the xenografts as detected by TUNEL (Terminal deoxynucleotidyl transferase dUTP nick end labeling assay) The brown color represents apoptotic cells. The sections were counterstained with methyl green hence the green color. **A.** L(-)ID4, **B.** L(-)ID4+NC, **C.** L(-)ID4+ID4NC, **D.** L(-)ID4+ DTX, **E.** L(-)ID4+NC+ DTX and **F.** L(-)ID4+ID4NC+ DTX. Representative images are shown. **G.** The apoptotic index (percent apoptotic cells) was calculated by counting the brown nuclei and dividing by total number of cells in a field (n=10 field). The data is mean±SEM (*p<0.01, ***p<0.001).

The degree of apoptosis in the xenografts was used to understand the mechanism of tumor regression in response to various treatments. Representative examples of apoptosis at the conclusion of the experiments are shown in Figure 6A-6F. The increase in the number of apoptotic nuclei in ID4NC group as compared to DTX, control and NC alone groups is clearly visible in the images (compare Figure 6C with 6A, 6B and 6D). The apoptotic index was significantly higher in xenografts receiving DTX as compared to untreated controls (Figure 6G, P<0.05). Interestingly, the apoptotic index in response of ID4-NC was approximately 3 fold higher as compared to DTX treatment (Figure 6G). The combination of DTX and ID4NC further increased the number of apoptotic cells by 1.5 fold as compared to ID4NC (P=0.04) and >5x as compared to DTX alone (P<0.001) (Figure 6G). Thus ID4-NC alone or in combination with DTX results in a significant increase in apoptosis in the L(-)ID4 cells *in vivo*.

### Immuno-histochemical analysis

**Figure 7:**
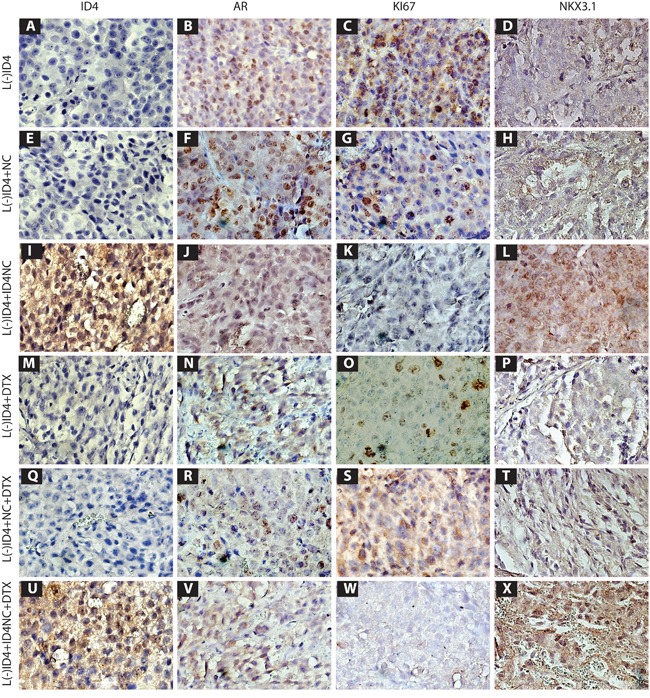
Immuno-histochemical localization (Brown staining) of ID4, AR, Ki67 and NKX3 1 in L(-)ID4, L(-)ID4+NC, L(-)ID4+ID4NC, L(-)ID4+ DTX, L(-)ID4+NC+ DTX and L(-)ID4+ID4NC+ DTX xenografts. Representative images from 3 different experiments are shown.

As shown in Figure 7I and 7U, ID4 expression was observed by IHC only in L(-)ID4+ ID4NC and L(-)ID4+ ID4NC+DTX tumors respectively and not in other tumors. ID4 localization in tumors receiving ID4NC was primarily nuclear although cytoplasmic staining was also observed. These results confirmed the successful delivery of recombinant ID4 to the tumors and demonstrated the specificity of the subsequent results.

Consistent with our earlier studies [4], AR expression was detected in all tumors albeit at various levels. As reported earlier, a predominantly nuclear AR expression was maintained in L(-)ID4 and L(-)ID4+NC xenografts (Figures 7B and 7F). In contrast, the expression of NKX3.1, an androgen induced [27] prostate tumor suppressor [28] was essentially undetectable in both control groups (Figure 7D and 7H). The expression of nuclear AR in ID4NC treated xenografts was more diffused and appeared to be lower (Figure 7J) as compared to the control groups (Figure 7B and 7F) whereas NKX3.1 expression used here as a marker of AR activity was the highest in xenografts receiving ID4NC (Figure 7L and 7X).

The AR positive nuclei in DTX treated xenografts (Figure 7N, 7R, 7V) was lower than respective controls however, NKX3.1 expression though detectable, was still significantly lower as compared to ID4NC (Figure 7L). Similar results were obtained when DTX was delivered in combination with NC alone (Figure 7R and 7T). In contrast, the AR expression appeared more diffused with a significant increase in NKX3.1 expression in the presence of both DTX and ID4-NC. These results suggested that ID4 promotes NKX3.1 expression.

## DISCUSSION

The biodegradable PCL and MD are biocompatible nano-carriers that demonstrate high cyto-compatibility with normal prostate epithelial and PCa cell lines [22]. In this study we demonstrate that ID4, a much smaller protein (∼18kd) as compared to BSA (∼66kD) was not only encapsulated but was also released in LNCaP cells *in vitro* and *in vivo* during active tumor growth. Functionally, the NC based delivery of recombinant ID4 is biologically active in terms of an anti-tumor activity and re-activation of tumor suppressor pathways that are relevant in PCa such as expression of PCa tumor suppressor NKX3.1 [28, 29].

Genetic ablation of ID4 in LNCaP cells (L(-)ID4) results in constitutively active AR (CRPC) and increased tumorigenicity (*in vivo and in vitro*) [4]. Consequently, re-constituting ID4 protein expression through ID4NC in L(-)ID4 reverses these effects suggesting specificity of ID4 tumor suppressor pathways such as increase in CDKNIs p27 and p21 [4]) and increased apoptosis following ID4NC treatment. Collectively, these multiple mechanisms could be involved in massive tumor regression *in vivo* following intratumoral NC mediated delivery of recombinant ID4.

Androgen receptor undergoes transition from a tumor suppressor in normal prostate to an oncogene in PCa [30], which is in part associated with re-programming of its transcriptional activity [31]. For example, AR in L(-)ID4 cells and prostate epithelial cells from Id4−/− mice fails to express NKX3.1 (a homeodomain protein), an androgen activated PCa tumor suppressor [27–29] [32] [6]. In contrast, NKX3.1 is re-expressed following treatment of L(-)ID4 xenografts with ID4NC, which is in part associated with a change in AR expression and localization. The tumorigenic AR activity during PCa progression and specifically in CRPC is dependent on multiple factors that include increased stability, post-translational modifications, interaction with specific co-regulators, and co-factors and ligand promiscuity. ID4 may regulate a specific or combination of these pathways that may ultimately decide whether AR acts as a tumor suppressor or an oncogene. Nevertheless, our results provide direct evidence that the constitutive oncogenic AR activity in CRPC is reversible, a significant observation. The underlying ID4 dependent molecular mechanisms could therefore be exploited to develop therapeutic strategies to re-activate AR as a tumor suppressor. Collectively, our results suggested that ID4NC may redirect AR activity from being a tumor promoter to that of a tumor suppressor.

Studies have demonstrated that loss of PTEN promotes prostate tumorigenesis [33, 34]. The LNCaP cells have a deletion of one allele and a mutation of the other *PTEN* allele but surprisingly are still not tumorigenic. Thus PTEN loss alone is not an absolute requirement for PCa progression towards castration resistance. The LNCaP cells acquire castration resistance phenotype only when grown for extended periods of time under androgen deprived conditions as exemplified by the isogenic C81 cell lines [35]. Interestingly, the ID4 promoter is also hyper-methylated in C81 cells [2]. Based on our results, loss of other genes, most notably ID4 together with PTEN appears to be a key mechanism involved in acquisition of castration resistance phenotype. Reconstitution of ID4 expression alone in the PTEN negative background (e.g. L(-)ID4) is sufficient to reverse the castration resistance phenotype as demonstrated by increased NKX3.1 expression in this study. The underlying mechanisms involved in ID4 dependent regulation of castration resistance are still not clear, but appears to be complex since Id4 is also required to maintain Pten expression as evident from the Id4−/− mice prostates [6].

DTX is the standard first line chemotherapy for advanced PCa [24, 25]. Unfortunately, patients treated with DTX inevitably develop resistance and relapse resulting in a very limited survival advantage [36]. Thus understanding the molecular basis of resistance and discovering therapeutic strategies/agents that can overcome the resistant pathways is urgently needed. In an effort to increase survival, combination DTX therapies that target androgen receptor pathway, angiogenesis, apoptosis and growth factors are currently under clinical trials [37]. The L(-)ID4 tumors also appeared at least partially resistant to DTX, analogous to DTX resistant, androgen insensitive C81 cells [38]. DTX inhibits mitosis (blocking tubulin de-polymerization [39]) promotes apoptosis (down-regulating bcl-_XL_ and bcl-2 [40]) and inhibit AR expression [41, 42] which is consistent with our results in L(-)ID4 treated xenografts. Tumor growth and IHC studies clearly demonstrates that ID4 promotes sensitivity to DTX treatment by significantly promoting apoptosis and blocking cell proliferation. These results also support earlier studies demonstrating that ID4 promotes sensitivity to Doxorubicin induced apoptosis in DU145 cells [8]. Although the mechanism of action of Doxorubicin is different (DNA intercalation) as compared to that of DTX, but collectively, these studies suggest ID4 expression may promote sensitivity to chemotherapeutic drugs, irrespective of their mechanism of action.

Mechanistically, how ID4 promotes multiple tumor suppressive pathways including sensitivity to chemotherapeutic agents is largely unknown. From our earlier studies it appears that ID4 dependent acetylation of p53 and subsequent activation of p53 dependent pathways such as apoptosis, senescence and proliferation could be one of the mechanisms [7].

ID4 may also act as inhibitor of inhibitor of DNA binding/differentiation protein ID1 through protein-protein interactions and subsequent neutralization of its biological activity [10]. Multiple studies have shown that increased ID1 expression in PCa [12] is associated with castration resistance and resistance to chemotherapeutic agents [43–45]. Since ID4 lacks a DNA binding domain hence most of its regulatory actions may involve interactions with other proteins. The major observation that needs to be further investigated is the ability of ID4 to revert the oncogenic AR in PCa back to normal AR activity which appears to be that of a tumor suppressor.

The study supports the feasibility of using NC encapsulated ID4 protein as a PCa therapeutic. We speculate that at the molecular level, ID4 may integrate multiple regulatory pathways for example epigenetic re-programming, integration of multiple AR co-regulators or signaling pathways that may ultimately result in tumor suppressor activity of ID4.

## MATERIALS AND METHODS

### Cell lines

Human PCa cell line LNCaP was obtained from ATCC and maintained at 37°C and 5% CO_2_ in RPMI-1640 supplemented with 10% (v/v) fetal bovine serum (FBS) and penicillin/streptomycin. ID4 was stably silenced in LNCaP cells using gene specific shRNA (L(-)ID4) as described earlier [7]. Non-silencing shRNA transfected LNCaP (L(-)shNS) was used as control.

### Recombinant ID4

Recombinant ID4 was expressed in E. Coli host strain BL21 (DE3) by transforming the pReceiver-B04 plasmid with GST tagged full length human ID4 (GeneCopeia Inc) as described previously [10].

### Preparation of ID4-loaded nanoparticles

The polycaprolactone (PCL)- maltodextrin (MD) encapsulated ID4 (ID4NC) was prepared as previously described [22] with modifications. RecombinantID4 in MD was used as the aqueous phase and a solution of PCL in methylene chloride as organic phase (Figure 1A). A 2% solution of polyvinyl alcohol (PVA) was used as a stabilizer. About 5 mg of ID4 protein was used for the formulation. Evaporation of the organic phase caused coacervation of the polymer and formation of a nano-suspension. The nano-suspension was centrifuged at 30,000g, washed at least three times with deionized water to remove any residual PVA or methylene chloride. The nanocarriers were characterized for their size and size distribution using Dynamic Light Scattering with a Malvern Zetasizer. The particle size, shape and morphology was confirmed by Scanning Electron Microscopy (Topcon DS-130F Field Emission Scanning Electron Microscope at Emory University).

### Cell cycle analysis and apoptosis assay

The cells were cultured in RPMI-1640 media without serum for 24hrs. Subsequently, the media was replaced with RPMI-1640 supplemented with 10% (v/v) fetal bovine serum (FBS). At this time, the cells either were left untreated (control) or supplemented with blank PCL-MD NC (NC) or NC loaded with recombinant ID4 (ID4NC). The cell cycle distribution was determined after 24 hours by staining DNA with propidium iodide (PI, Calbiochem). Apoptosis was quantitated by staining with PI and Alexa Fluor 488 conjugated Annexin V (Molecular Probes). The PI or PI+Annexin V cells were analyzed with Accuri C6 flow cytometer.

### Transwell migration and soft agar colony forming assay

Cell migration was performed using Boyden chambers according to the manufacturer's protocol (BD, Bedford MA as described previously [4].

For the soft agar colony forming assay, L(-)ID4 cells along with NC or ID4NC (10^4^ cells/well) were suspended in 0.3% Difco Noble agar (Difco, Detroit, MI) supplemented with complete RPMI 1640 medium with 20% FBS. This suspension was layered over 0.8% agar base layer in 6 well plates. The plates were incubated at 37°C with 5% CO_2_ in a humidified incubator and fresh medium was overlaid every 3 days. The colony numbers were counted using a microscope after 2-3 weeks of incubation.

### Immuno -cytochemistry (ICC) and –histochemistry (IHC)

ICC and IHC protein localization studies on cells grown in glass chamber slides or paraffin embedded 5um tissue sections respectively was performed as described earlier [4, 12]. Non-immune IgG was used as control for all immune localization studies and resulted in lack of detection of the antigens (data not shown).

### Immuno blot analysis

Total cellular proteins were prepared from cultured cells using M-PER (Thermo Scientific). Immuno-blot analysis using protein specific antibodies were performed as described earlier [6] The LAS 3000 imager (Fuji) and image quant software was used to capture and quantify the images.

### Animal studies

All studies were approved by the Clark Atlanta University and Mercer University committee for the use and care of animals. L(-)ID4 cells (2×10^6^) suspended in 100 μL of serum-free RPMI 1640 medium containing Matrigel (1:1 [v/v], BD Biosciences) were injected subcutaneously in 4-week-old male SCID Beige mice (Taconic Biosciences, Inc.). After 6 weeks of injection, the mice were divided into six different groups. The first group received no treatments while the second and the third group received intratumoral injections of NC and ID4NC, respectively twice a week. The fourth, fifth and sixth groups received similar treatments as the above three groups along with intraperitoneal injections of the Docetaxel (DTX) (5 mg/kg body weight, twice weekly). The growth of the tumors was measured each week using digital calipers and the volume was calculated. At the end of four weeks of treatments, tumors were surgically removed, weighed and the volume was measured. Harvested tumors were fixed in 10% buffered formalin. The fixed tumors were paraffin embedded, sectioned and stained with either hematoxylin and eosin or used for IHC. Images were captured using a Zeiss microscope with an AxiomCam 4.5 imaging system.

### TUNEL assay

Apoptosis in paraffin-embedded sections of the xenografts were studied by terminal deoxynucleotidyl transferase-mediated dUTP nick-end-labeling (TUNEL) assay. Staining was carried out according to the protocol provided by the supplier (GeneTex). The number of TUNEL positive cells were divided by the total number of nuclei in five random fields in order to determine the apoptotic index.

### Data and statistical analysis

The NIH Image J [46] was used for counting cells stained positive for respective antigens in IHC studies [6]. Within group Student's t-test was used for evaluating the statistical differences between groups.

## SUPPLEMENTARY MATERIALS FIGURE


