# Supplementary material for: Intra-tumoral delivery of functional ID4 protein via PCL/maltodextrin nano-particle inhibits prostate cancer growth
Source: Oncotarget. 2016 Jul 30;7(42):68072–85. doi: 10.18632/oncotarget.10953 (PMC5340093; doi:10.18632/oncotarget.10953)
Supplement: Supplementary file 1 [file oncotarget-07-68072-s001.pdf]

Intra-tumoral delivery of functional ID4 protein via PCL/maltodextrin nano-particle inhibits prostate cancer growth

SUPPLEMENTARY FIGURE

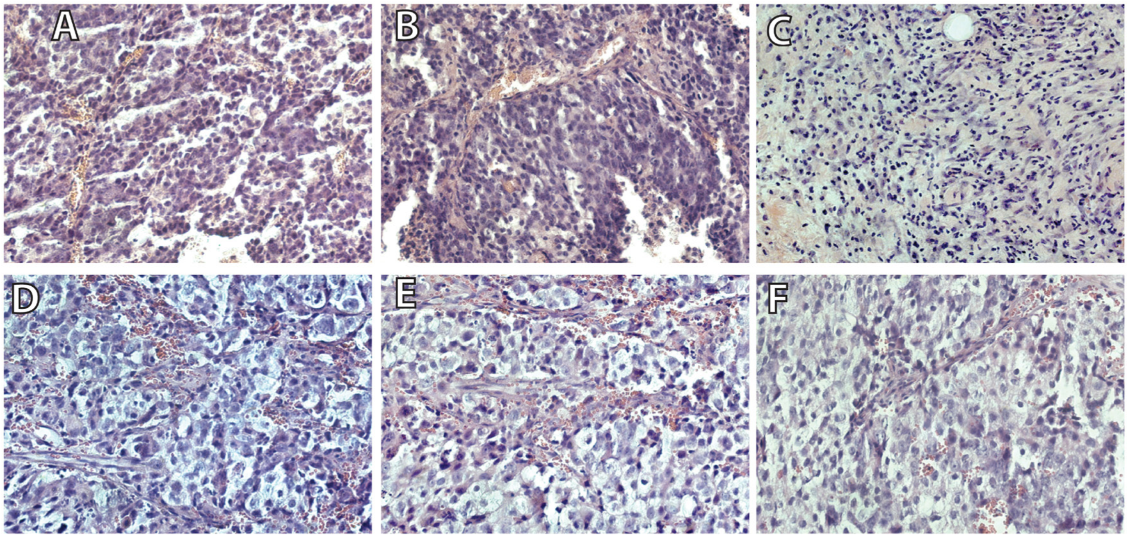

Supplementary Figure S1: Histological l analysis of tumors following excision. The 5um sections were stained with H&E. **A.** L(-)ID4, **B.** L(-)ID4+NC, **C.** L(-)ID4+ID4NC, **D.** L(-)ID4+ DTX, **E.** L(-)ID4+NC+ DTX and **F.** L(-)ID4+ID4NC+ DTX. Representative images are shown.
